# Melatonin Suppresses Renal Cortical Fibrosis by Inhibiting Cytoskeleton Reorganization and Mitochondrial Dysfunction through Regulation of miR-4516

**DOI:** 10.3390/ijms21155323

**Published:** 2020-07-27

**Authors:** Yeo Min Yoon, Gyeongyun Go, Chul Won Yun, Ji Ho Lim, Jun Hee Lee, Sang Hun Lee

**Affiliations:** 1 Medical Science Research Institute, Soonchunhyang University Seoul Hospital, Seoul 04401, Korea; yoonboo15@naver.com (Y.M.Y.); skydbs113@naver.com (C.W.Y.); wlenfl1@naver.com (J.H.L.); jhlee0407@sch.ac.kr (J.H.L.); 2 Department of Biochemistry, College of Medicine, Soonchunhyang University, Cheonan 31151, Korea; ggy0227@naver.com

**Keywords:** melatonin, miR-4516, renal cortical fibrosis, TH1 cells, cytoskeleton reorganization

## Abstract

Renal fibrosis, a major risk factor for kidney failure, can lead to chronic kidney disease (CKD) and is caused by cytoskeleton reorganization and mitochondrial dysfunction. In this study, we investigated the potential of melatonin treatment to reduce renal fibrosis by recovering the cytoskeleton reorganization and mitochondrial dysfunction. We found that miR-4516 expression was downregulated in the renal cortex of CKD mice and *P*-cresol-treated TH1 cells. Decreased miR-4516 expression stimulated cytoskeleton reorganization and mitochondrial dysfunction, and induced renal fibrosis. Melatonin treatment suppressed fibrosis by inhibiting cytoskeleton reorganization and restoring mitochondrial function via increased miR-4516 expression. More specifically, melatonin treatment increased miR-4516 expression while decreasing ITGA9 expression, thereby inhibiting cytoskeleton reorganization. In addition, increased expression of miR-4516 by melatonin treatment reduced ROS formation and restored mitochondrial function. These findings suggest that melatonin may be a promising treatment for patients with CKD having renal fibrosis. Moreover, regulation of miR-4516 expression may be a novel strategy for the treatment of renal fibrosis.

## 1. Introduction

Chronic kidney disease (CKD) presented a global all-age mortality rate of 45.5% between 1990 and 2017. CKD is defined as a gradual loss of kidney function and/or structure over a period of months to years [1,2]. In patients with CKD, kidney dysfunction is mainly caused by progressive fibrosis. Although fibrosis is a damage repair response in connective tissues, an excess accumulation of the extracellular matrix results in loss of functional tissue when normal tissue is replaced [3,4]. Previous reports have shown that cellular damages, such as cytoskeleton reorganization, and mitochondrial dysfunction causes kidney fibrosis [5,6]. Activation of cytoskeleton reorganization induces the expression of fibrosis-related proteins, such as fibronectin and collagen type 1. In addition, mitochondrial dysfunction induces renal fibrosis by inducing ROS generation, NLRP3 inflammasome activation, and pro-inflammatory cytokines: IL-1β, and IL-18 [7]. Therefore, it is important to inhibit cytoskeleton reorganization and mitochondrial dysfunction to suppress renal fibrosis.

MicroRNAs (miRNAs) are short noncoding RNAs which are 21–25 nucleotides in length, which play important roles in regulating various cellular and physiological processes by binding to the 3′-untranslated region (UTR) of the target mRNA to alter its expression [1,8]. Recently, functional studies have shown that miRNAs can act as critical modulators of certain diseases [9]. MiR-4516 is known to be associated with autophagy [10], and overexpression of miR-4516 is known to increase proliferation and invasion of glioblastomas [11]. Importantly, miR-4516 regulates the expression of fibronectin 1 and ITGA9, a regulator of cytoskeleton reorganization, and inhibits keratinocyte motility [12]. In addition, miR-4516 suppress mitochondrial dysfunction by inhibiting mitochondrial ROS generation [13]. Therefore, by regulating the expression of miR-4516, cytoskeleton reorganization and mitochondrial function can be restored.

Melatonin is the main hormone synthesized from serotonin and is secreted by the pineal gland at night. Melatonin is associated with several physiological functions, such as sleep, circadian rhythms, and neuroendocrine actions [13,14]. Accumulating studies have also shown that melatonin can facilitate therapeutic functional recovery in myocardial infarction, skin lesions, pulmonary ischemia repression injury, and sepsis-induced kidney injury [13,15,16]. Importantly, melatonin is known to restore miRNA expression, especially miRNA-4516 in mesenchymal stromal/stem cells (MSCs) from patients with CKD and increased therapeutic efficacy of MSCs [13]. Therefore, we investigated whether melatonin could reduce renal fibrosis in the *P*-cresol-treated renal proximal tubule epithelial cells and in the CKD mouse model by regulating miR-4516.

## 2. Results

### 2.1. miR-4516 Is Downregulated in the Kidney Cortex of the CKD Mouse Model

**Figure 1:**
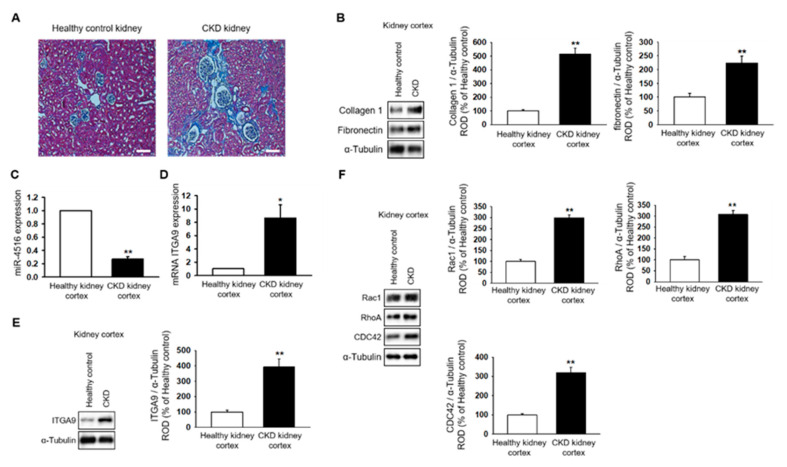
Expression of miR-4516 is downregulated in renal cortical fibrosis in a CKD mouse model. (**A**) In healthy mice (*n* = 3) or CKD mice (*n* = 3), hematoxylin and eosin (H&E) staining was performed on kidney sections (scale bar = 1000 μm). (**B**) Western blot analysis for collagen type 1, and fibronectin in the kidney cortex of healthy mice and CKD mice (*n* = 3). Protein expression was determined by densitometry relative to α-tubulin. The values represent mean ± SEM. ** *p* < 0.01 vs. healthy kidney cortex. (**C**,**D**) Expression of miR-4516 and ITGA9 was detected in the kidney cortex of healthy mice (*n* = 3) or CKD mice (*n* = 3) by quantitative real-time polymerase chain reaction (qPCR). The values represent mean ± SEM. * *p* < 0.05, ** *p* < 0.01 vs. healthy kidney cortex. (**E**,**F**) Western blot analysis for ITGA9, Rac1, RhoA, and CDC42 in the kidney cortex of healthy mice and CKD mice (*n* = 3). Protein expression was determined by densitometry relative to α-tubulin. The values represent the mean ± SEM. ** *p* < 0.01 vs. healthy kidney cortex.

To establish the CKD mouse model, BALB/c nude mice were fed 0.75% adenine for 1–2 weeks. The CKD mouse model was confirmed by the increased glomerulus size and expression of fibrosis-associated proteins in the kidneys (Figure 1A,B). Next, we used qPCR to show that miR-4516 expression is significantly downregulated in the kidney cortex of CKD mice (Figure 1C). We hypothesized that if miR-4516 expression was reduced, ITGA9 expression would increase leading to cytoskeleton reorganization. Indeed, CKD kidneys showed increased ITGA9 expression compared to a healthy kidney cortex (Figure 1D,E). To confirm activation of cytoskeleton reorganization, we measured the expression of GTPase signaling proteins, such as Rac1, RhoA, and CDC42, in the fibrotic area of the renal cortex. We found that Rac1, RhoA, and CDC42 expression was significantly upregulated in the kidney cortex of CKD mice (Figure 1F). These data indicate that renal fibrosis is associated with decreased miR-4516 expression and increased ITGA9 signaling, which initiates stress fiber actin signaling.

### 2.2. Melatonin Reduces P-Cresol-Induced Activation of Cytoskeleton Reorganization and Fibrosis via Increased miR-4516 Expression

**Figure 2:**
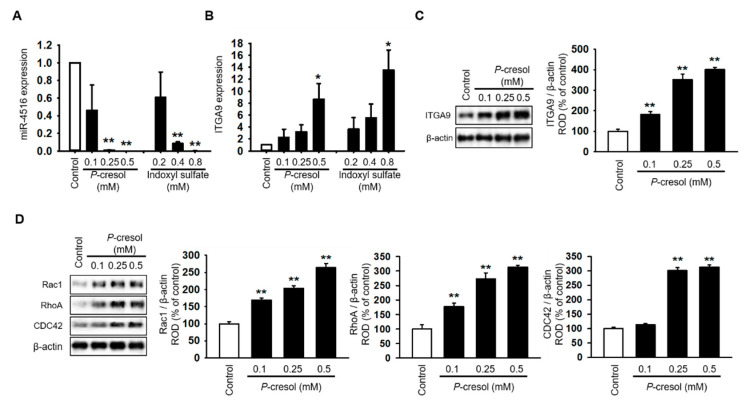
Cytoskeleton reorganization and ITGA9-Rho GTPase signaling pathways are activated due to decreased miR-4516 expression following *P*-cresol exposure. (**A**,**B**) Expression of miR-4516 and ITGA9 was detected in human proximal tubular epithelial (TH1) cells with *P*-cresol (0.1, 0.25, and 0.5 mM) or indoxyl sulfate (0.2, 0.4, and 0.8 mM) exposure for 72 h (*n* = 3). The values represent mean ± SEM. * *p* < 0.05, ** *p* < 0.01 vs. control. (**C**,**D**) Western blot analysis for ITGA9, Rac1, RhoA, and CDC42 in TH1 cells after exposure to various doses of *P*-cresol (0, 0.1, 0.25, and 0.5 mM) for 72 h (*n* = 3). Protein expression was determined by densitometry relative to β-actin. The values represent mean ± SEM. ** *p* < 0.01 vs. control.

**Figure 3:**
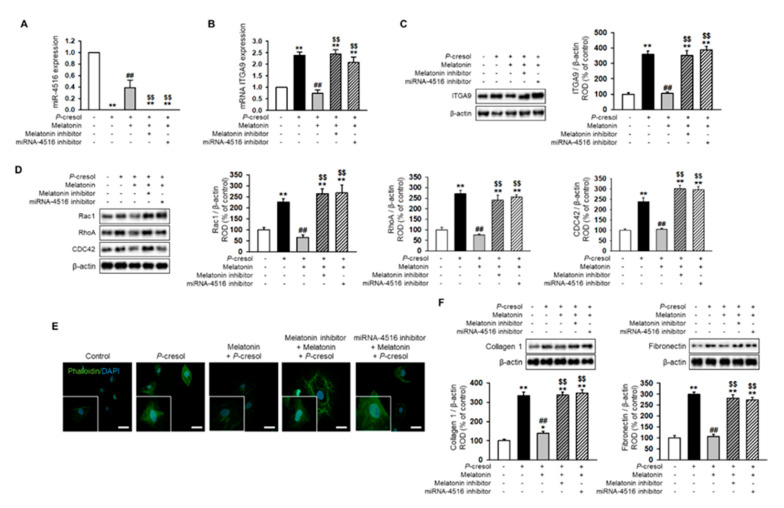
Melatonin reduces cytoskeleton reorganization and expression of fibrosis markers via increased expression of miR-4516. (**A**,**B**) Expression of miR-4516 and ITGA9 was detected in melatonin-treated TH1 cells (1 μM for 24 h) under *P*-cresol exposure (0.5 mM for 72 h). TH1 cells were pre-treated with the miR-4516 inhibitor (200 nM) or luzindole (melatonin inhibitor, 1 μM) for 48 h and then treated with melatonin (*n* = 3). The values represent mean ± SEM. ** *p* < 0.01 vs. control, ^##^ *p* < 0.01 vs. *P*-cresol exposure condition, ^$$^ *p* < 0.01 vs. melatonin-treated TH1 cells in *P*-cresol exposure condition. (**C**,**D**) Western blot analysis for ITGA9, Rac1, RhoA, and CDC42 in melatonin-treated TH1 cells (1 μM for 24 h) or pre-treated with the miR-4516 inhibitor (200 nM for 48 h) or luzindole (1 μM for 48 h) under *P*-cresol exposure (0.5 mM for 72 h) (*n* = 3). Protein levels were determined by densitometry relative to that of β-actin. The values represent mean ± SEM. ** *p* < 0.01 vs. control, ^##^ *p* < 0.01 vs. *P*-cresol exposure condition, ^$$^ *p* < 0.01 vs. melatonin-treated TH1 cells in *P*-cresol exposure condition. (**E**) Phalloidin staining was performed in melatonin-treated TH1 cells (1 μM for 24 h) or pre-treated with the miR-4516 inhibitor (200 nM for 48 h) or luzindole (1 μM for 48 h) in exposure to *P*-cresol (0.5 mM for 72 h) (scale bar = 40 μm, *n* = 3). (**F**) Western blot analysis for collagen type 1 and fibronectin in melatonin-treated TH1 cells (1 μM for 24 h) or pre-treated with the miR-4516 inhibitor (200 nM for 48 h) or luzindole (1 μM for 48 h) under *P*-cresol exposure (0.5 mM for 72 h) (*n* = 3). Protein levels were determined by densitometry relative to that of β-actin. The values represent mean ± SEM. ** *p* < 0.01 vs. control, ^##^ *p* < 0.01 vs. *P*-cresol exposure condition, ^$$^ *p* < 0.01 vs. melatonin-treated TH1 cells in *P*-cresol exposure condition.

Treatment with *P*-cresol or indoxyl sulfate induced a gradual decrease in miR-4516 expression and an increase in RNA and protein expression of ITGA9 in TH1 cells in a dose-dependent manner (Figure 2A–C). To confirm that *P*-cresol treatment induces activation of actin remodeling by expression of ITGA9, we measured the expression of Rho GTPase signaling proteins using Western blot analysis. We found that Rac1, RhoA, and CDC42 expression also increased in *P*-cresol-treated TH1 cells in a dose-dependent manner (0, 0.1, 0.25, and 0.5 mM; Figure 2D). Based on these results, we hypothesized that by increasing the expression of miR-4516, we could reduce ITGA9 expression and inhibit the activation of actin remodeling to suppress fibrosis. Previous studies have shown that melatonin treatment increases miR-4516 expression [13]. Therefore, we assumed that melatonin treatment could suppress ITGA9 expression and, thus, inhibit renal fibrosis. Treatment of TH1 cells with melatonin markedly increased miR-4516 expression (Figure 3A) and decreased ITGA9 expression at the mRNA and protein level (Figure 3B,C). Next, we measured the expression of Rho GTPase signaling proteins and stress fiber actin (F-actin) using phalloidin staining to confirm that melatonin inhibits cytoskeleton reorganization and fibrosis in *P*-cresol-treated TH1 cells. Our data show that melatonin treatment significantly decreases the expression of Rho GTPase signaling proteins and stress fiber actin in *P*-cresol-treated TH1 cells (Figure 3D,E). In addition, treatment with melatonin inhibitors and the miRNA-4516 inhibitor abolishes these melatonin-induced effects (Figure 3D,E). Treatment of TH1 cells with melatonin also decreases the expression of fibrosis-associated proteins such as collagen type 1 and fibronectin (Figure 3F). Thus, these data suggest that melatonin treatment protects renal proximal tubular cells against cytoskeleton reorganization and fibrosis by increasing miR-4516 expression.

### 2.3. Melatonin Treatment Restores Mitochondrial Dysfunction via Increased miR-4516 Expression

**Figure 4:**
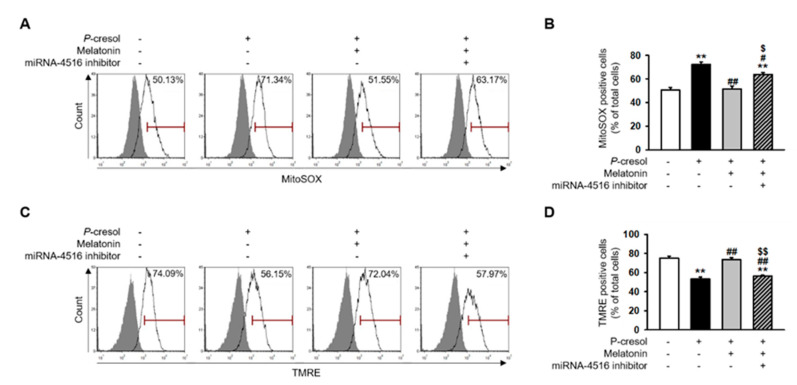
Melatonin inhibits mitochondrial ROS generation and restores mitochondrial membrane potential via increased expression of miR-4516. (**A**) Flow cytometry analysis of MitoSOX in melatonin-treated TH1 cells (1 μM for 24 h) or cells pre-treated with the miR-4516 inhibitor (200 nM for 48 h) under *P*-cresol exposure (0.5 mM for 72 h) (*n* = 3). (**B**) Quantification of the number of MitoSOX-positive cells. (**C**) Flow cytometry analysis of TMRE in melatonin-treated TH1 cells (1 μM for 24 h) or cells pre-treated with the miR-4516 inhibitor (200 nM for 48 h) under *P*-cresol exposure (0.5 mM for 72 h) (*n* = 3). (**D**) Quantification of the number of TMRE-positive cells. The values represent mean ± SEM. ** *p* < 0.01 vs. control, ^#^ *p* < 0.05, ^##^ *p* < 0.01 vs. *P*-cresol exposure condition, ^$^ *p* < 0.05, ^$$^ *p* < 0.01 vs. melatonin-treated TH1 cells following *P*-cresol exposure.

**Figure 5:**
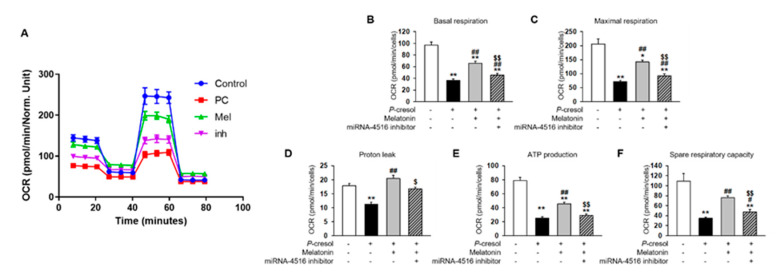
Melatonin restores mitochondrial function via increased expression of miR-4516. (**A**) Oxygen consumption rate (OCR) of each group was measured over time (min). The mitochondrial OCR was used to obtain bioenergetic parameters by adding oligomycin (20 min, 1 μM), FCCP (40 min, 0.75 μM), and antimycin A (60 min, 1 μM). (**B**) The non-mitochondrial OCR was subtracted to obtain the basal mitochondrial OCR (remaining OCR after antimycin A addition). (**C**) FCCP was used to obtain the maximal respiration OCR. (**D**) Proton leak was calculated using the difference between OCR following oligomycin A inhibition and OCR following antimycin A inhibition. (**E**) ATP production was determined using the difference between the basal and antimycin A inhibited OCR. (**F**) Spare respiratory capacity was calculated using the difference between the OCR following oligomycin A inhibition and the OCR following FCCP treatment. The histogram shows representative data from one replicate experiment (*n* = 5). The values represent mean ± SEM. * *p* < 0.05, ** *p* < 0.01 vs. control, ^#^ *p* < 0.05, ^##^ *p* < 0.01 vs. *P*-cresol exposure condition, ^$^ *p* < 0.05, ^$$^ *p* < 0.01 vs. melatonin-treated TH1 cells in following *P*-cresol exposure.

In previous studies, *P*-cresol treatment increased mitochondrial reactive oxygen species (ROS) and decreased mitochondrial membrane potential and induced mitochondrial dysfunction [17]. Here, we found that melatonin treatment markedly inhibits mitochondrial ROS generation in *P*-cresol-treated TH1 cells (Figure 4A,B). In addition, melatonin treatment restores mitochondrial membrane potential in *P*-cresol-treated TH1 cells (Figure 4C,D). However, treatment with the miR-4516 inhibitor or melatonin inhibitor blocks these effects (Figure 4A–D, Figure S1A–D). We further investigated the protective effects of melatonin on mitochondrial function using a seahorse analyzer. The results showed that melatonin treatment protected TH1 cells against a *P*-cresol-induced decrease in oxygen consumption rate (OCR)(Figure 5A–F). Treatment with the miR-4516 inhibitor or melatonin inhibitor blocks these effects (Figure 5A–F, Figure S2A–F). Thus, the restorative effect of melatonin prove that the OCR is amplified via miR-4516 expression. We also confirmed the protective effect of melatonin on cell cycle and cellular apoptosis in *P*-cresol-treated TH1 cells. Melatonin treatment restores *P*-cresol-induced changes in the cell cycle and in cellular apoptosis (Figure S2A–D). These results suggest that melatonin restores *P*-cresol-induced mitochondrial and cellular dysfunction via increased expression of miR-4516.

### 2.4. Melatonin Injection Suppresses Renal Fibrosis in a CKD Mouse Model

**Figure 6:**
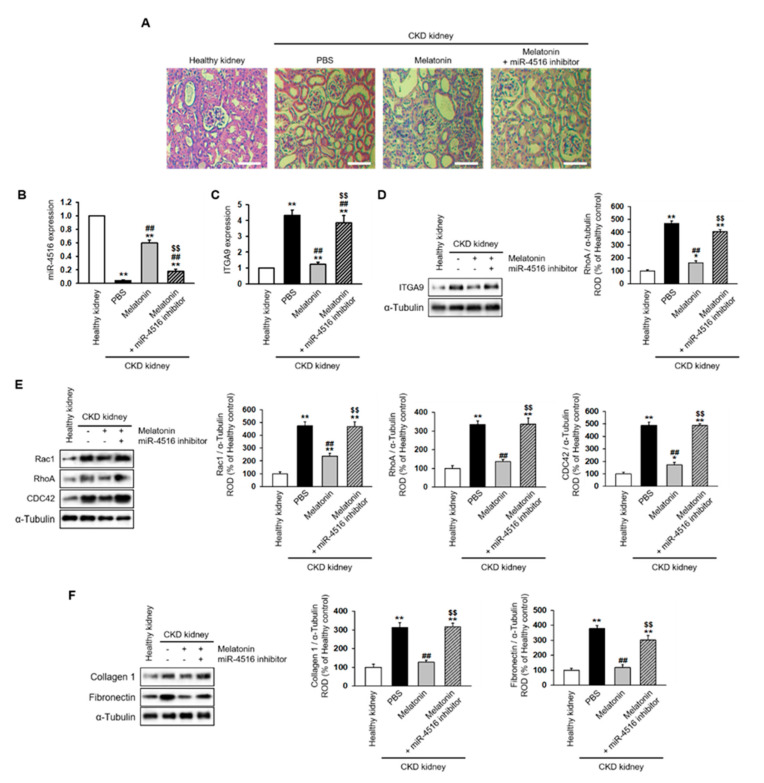
Melatonin injection restores renal cortical fibrosis in a CKD mouse model via increased expression of miR-4516. (**A**) Hematoxylin and eosin (H&E) staining was performed on kidney sections from a CKD mouse model following melatonin injection, or melatonin inhibition with miR-4516 inhibitor (scale bar = 1000 μm). (**B**,**C**) Expression of miR-4516 and ITGA9 was detected in the kidney cortex in each group by qPCR (*n* = 3). (**D**–**F**) Western blot analysis for ITGA9, Rac1, RhoA, CDC42, collagen type 1, and fibronectin expression using samples from the kidney cortex of each mouse model group (*n* = 3). Protein levels were determined by densitometry relative to α-tubulin. The values represent mean ± SEM. * *p* < 0.05, ** *p* < 0.01 vs. healthy kidney, ^##^ *p* < 0.01 vs. phosphate buffered saline (PBS), ^$$^ *p* < 0.01 vs. melatonin.

To understand whether melatonin can suppress renal fibrosis, melatonin was intraperitoneally injected into CKD mice twice per week for two weeks. The mice were then sacrificed for detection of renal fibrosis using H&E staining. The data show that melatonin injection markedly suppresses renal fibrosis in the CKD mouse model (Figure 6A). In addition, co-treatment with the miR-4516 inhibitor blocked the effect of melatonin on renal fibrosis (Figure 6A). Moreover, melatonin injections restored miR-4516 expression while suppressing mRNA and protein expression of ITGA9 in CKD kidneys. Treatment with miR-4516 inhibitor abolished these effects (Figure 6B–D). We also measured Rho GTPase signaling proteins and fibrosis-related proteins in CKD kidneys. Interestingly, melatonin injections restored the changes in the GTPase signaling pathway and suppressed the expression of collagen type 1 and fibronectin (Figure 6E,F). These results show that melatonin recovers renal fibrosis by increasing miR-4516 expression.

## 3. Discussion

Renal disorder pathogenesis is led by progressive fibrosis, which is caused by an excessive repair response in the damaged tissues of patients with CKD. The renal cortex is composed of various types of cells that constitute the tubules, interstitium, and capillaries. Thus, dysfunction of the renal cortex with fibrosis attenuates the major kidney function of ultrafiltration. In this study, we demonstrated that miR-4516 expression is downregulated in CKD mice, and that renal fibrosis is suppressed when miR-4516 expression is restored by melatonin treatment. These results suggest that miR-4516 is a promising therapeutic target for renal fibrosis.

Recently, miRNAs, which regulate various cellular signaling and physiological processes by binding to the target mRNA, have been shown to be important disease modulators, and potential therapies [9,18,19]. For example, miR-21 is known to induce renal fibrosis via silencing of metabolic pathways [20]. In our previous studies, we identified other causes of renal fibrosis, such as stress fiber actin (F-actin), mitochondrial dysfunction, and cellular senescence [13,14,17]. Importantly, we found that decreased miR-4516 expression in MSCs isolated from patients with CKD (CKD-MSCs) induced cellular senescence, mitochondrial dysfunction, and reduced proliferative potential [13]. The miR-4516 sequence is contained in the polycystin kidney disease 1 (PKD1) sequence which is found in 16 chromosomes and consists of 46 exons [21]. Prior studies have shown that mutations in PKD1 correlate with various renal diseases; therefore, mutations in PKD1 might reduce miR-4516 expression [22,23]. Our data also show that miR-4516 expression is downregulated in the kidney cortex of CKD mice. 

In this study, we have shown that the expression of ITGA9, a miR-4516 target, is upregulated in the CKD mouse model. ITGA9 increases cytoskeleton reorganization signaling by increasing the Rho GTPases associated proteins, Rac1, RhoA, and CDC42 [12]. These signaling events increase the expression of the fibrosis marker proteins, fibronectin and collagen type 1 [24]. Our data showed similar cell signaling pathways to fibrosis via decreased expression of miR-4516. In vitro data also demonstrated that human proximal tubular epithelial (TH1) cells show decreased expression of miR-4516 following *P*-cresol exposure. Thus, Rho GTPase-associated proteins increased stress fiber actin (F-actin)- induced fibrosis transcriptional factors under conditions of *P*-cresol exposure [24]. These results suggest that increased miR-4516 expression may reduce activation of renal fibrosis by decreasing cytoskeleton reorganization signaling.

CKD is known to induce mitochondrial dysfunction by increasing mitochondrial ROS generation and, thus, reducing mitochondrial activity [13]. Similarly, *P*-cresol induces mitochondrial dysfunction by inducing ROS generation, mitofusion, and reducing mitophagy [17]. Our previous studies indicated that melatonin enhanced mitochondrial function under *P*-cresol exposure by increasing catalase activity and decreasing mitochondrial ROS generation [25]. Although these results proved that melatonin had similar effects on restored mitochondria, we elucidated the mechanism by which increased miR-4561 expression regulated mitochondrial function.

The effects of melatonin can be mediated by its interaction with melatonin receptors type 1 and type 2 (MT1 and MT2, respectively) or intracellular receptors [26]. Melatonin receptors (MT1 and MT2) are known to be expressed in the renal cortical epithelium of guinea pig and in the human fetal kidney cortex [27,28]. In this study, the effects of melatonin were inhibited by luzindole, a selective melatonin receptor antagonist. These results indicate that the protective effects of melatonin were mainly achieved through melatonin receptors.

Previous studies have shown that that cytoskeleton could regulate morphology and function of the mitochondria [29]. In yeast, decreased actin turnover, which leads to the accumulation of F-actin aggregates, increases ROS production in mitochondria [30]. In the fibrosarcoma cell line L929, beta-actin is required for TNF-induced mitochondria clustering and ROS generation in mitochondria [31]. Therefore, it is possible that melatonin may restore the mitochondria functions by regulating cytoskeleton reorganization.

Herein, we present in vivo data showing that intraperitoneal injection of melatonin directly decreased CKD fibrosis and glomerulus swelling, leading to restored kidney function in a mouse model of CKD. Melatonin injections also resulted in increased miR-4516 expression in the kidney cortex. Thus, the melatonin-induced effect on miR-4516 expression reduced renal cortical fibrosis by suppressing cytoskeleton reorganization signaling, and attenuated ITGA9-Rho GTPase signaling activation.

In this study, we demonstrated that miR-4516 expression is reduced in the renal cortex of CKD mice and *P*-cresol-treated TH1 cells. Decreased miR-4516 expression induced activation of cytoskeleton reorganization, mitochondrial dysfunction, and renal fibrosis. Melatonin treatment suppressed fibrosis by inhibiting cytoskeleton reorganization and restoring mitochondrial function by increasing miR-4516 expression. In particular, melatonin treatment increased miR-4516 expression and decreased ITGA9 expression, thereby inhibiting cytoskeleton reorganization. In addition, we also confirm that increased miR-4516 expression following melatonin treatment reduced ROS formation, increased mitochondrial membrane potential, and restored mitochondrial function. These results suggest that melatonin may be a therapeutic agent for patients with CKD with renal fibrosis, and that the regulation of miR-4516 expression may be a promising novel strategy for the treatment of renal fibrosis.

## 4. Materials and Methods

### 4.1. Culture of Human Proximal Tubular Epithelial (TH1) Cells

TH1 cells were obtained from the American Type Culture Collection (Manassas, VA, USA). Cells were cultured in minimum essential medium (Gibco BRL, Gaithersburg, MD, USA) supplemented with 10% (*v*/*v*) fetal bovine serum (FBS; Gibco BRL) and 100 U/mL penicillin/streptomycin (Gibco BRL). The cells were grown in a humidified 5% CO2 incubator at 37 °C.

### 4.2. Transfection of miRNA Inhibitor

For inhibition of miR-4516, cells were transfected with miR-4516 inhibitor (200 nM) using Lipofectamine 2000 reagent (Thermo Fisher Scientific, Waltham, MA, USA) following the manufacture’s instruction. Reverse transcription-quantitative polymerase chain reaction analysis was performed to confirm successful transfection. Inhibitor of miR-4516 was purchased from abm Inc. (Richmond, BC, Canada).

### 4.3. Quantification of miRNA and ITGA9 mRNA

Total RNA (DNase digested) was isolated from TH1 cells or kidney cortex tissue preparations using the miRNeasy Mini Kit (Qiagen, Hilden, Germany). Quantitative real-time polymerase chain reactions (qRT-PCR) were performed using the TaqMan Small RNA Assay (Thermo Fisher Scientific) to determine miRNA expression. Expression levels were normalized to U6 rRNA or β-actin.

### 4.4. Western Blotting

Protein extracts from TH1 cells or kidney cortex tissue were separated via 8–12% sodium dodecyl sulfate-polyacrylamide gel electrophoresis and then transferred to polyvinylidene difluoride membranes. After blocking membranes with 5% skimmed milk for 1 h, primary antibodies against ITGA9, Ras-related C3 botulinum toxin substrate 1 (Rac1), Ras homolog family member A (RhoA), cell division control protein 42 homolog (CDC42), collagen type 1, fibronectin, cyclin-dependent kinase 2 (CDK2), cyclin E, cyclin-dependent kinase 4 (CDK4), cyclin D1, B-cell lymphoma 2 (BCL2), Bcl-2-associated X protein (BAX), cleaved caspase-3, cleaved poly (ADP-ribose) polymerase 1 (cleaved PARP1), α-tubulin and β-actin, were incubated overnight at 4 °C. After washing with phosphate buffered saline (PBS), the membranes were incubated with goat anti-rabbit IgG or goat anti-mouse IgG conjugated to horseradish peroxidase (Santa Cruz Biotechnology, Santa Cruz, CA, USA). The bands were detected by enhanced chemiluminescence (ECL; Amersham Pharmacia Biotech, England, UK). The mouse anti-α-tubulin (SC-8035), mouse anti-β-actin (SC-47778), mouse anti-BCL2 (SC-7382), rabbit anti-cleaved caspase-3 (SC-7148), mouse anti-CDK2 (SC-6248), mouse anti-CDK4 (SC-56277), mouse anti-cleaved PARP1 (SC-56196), mouse anti-cyclin D1 (SC-20044), rabbit anti-Bax (SC-6236), and mouse anti-cyclin E (SC-377100) antibodies were purchased from Santa Cruz Biotechnology. The rabbit anti-CDC42 (NBP2-68888), rabbit anti-RAC1 (NB100-91266), rabbit anti-ITGA9, and rabbit anti-RhoA (NB100-91273) antibodies were obtained from Novus Biologicals (Littleton, CO, USA). The mouse anti-fibronectin antibody (MA5-12314) was purchased from Thermo Fisher Scientific. The rabbit anti-collagen type 1 antibody (ab21286) was purchased from abcam (Cambridge, UK).

### 4.5. Phalloidin Staining

Phalloidin staining (Thermo Fisher Scientific) was performed for each TH1 cell group. Nuclei were stained with 4′,6-diaminido-2-phenylindol (DAPI; Sigma-Aldrich, St. Louis, MO, USA), and the immunostained samples were examined by confocal microscopy (Olympus, Tokyo, Japan).

### 4.6. Measurements of the Oxygen Consumption Rate (OCR)

The OCR was measured in TH1 cells using the XF96 Extracellular Flux analyzer (Seahorse Bioscience, Billerica, MA, USA). Briefly, TH1 cells were seeded at 4.5 × 104 cells in each well in XF96 cell culture multi-well plates and incubated for 24 h at 37 °C in a 5% CO_2_ incubator. XF96 cartridges were then incubated overnight in XF calibrant at 37 °C in a non-CO_2_ incubator. TH1 cell growth media was exchanged with XF media and incubated at 37 °C in a non-CO_2_ incubator for 1 h. Next, inhibitors were diluted to the appropriate concentrations in XF medium and loaded into the corresponding microwells in the XF96 cartridge plate. Following equilibration of the sensor cartridges, the XF96 cell culture plates were loaded into the XF96 Extracellular Flux analyzer at 37 °C, and the OCR was measured after cycles of mixing and acquiring data (basal) or inhibitor injection, mixing, and data acquisition. OCR was measured using Seahorse Wave Desktop Software (Agilent Technologies, Santa Clara, CA, USA).

### 4.7. Fluorescence Staining for Flow Cytometry

Each TH1 cell group was trypsinized and treated with 5 μM MitoSOX (Thermo Fisher Scientific) and 100 nM tetramethylrhodamine, methyl ester, and perchlorate (TMRE; Thermo Fisher Scientific) for 30 min. After washing twice with PBS, the samples were measured by flow cytometry (Sysmex, Kobe, Japan). MitoSOX- and TMRE positive cells were identified using FCS Express 5 Flow research software (De Novo Software, Los Angeles, CA, USA).

### 4.8. CKD Mouse Model

Eight-week-old male BALB/c mice were fed an adenine-containing diet (0.75% adenine) for 1–2 weeks. Body weight was measured every week, and the mice were randomly assigned to different groups each with 10 mice. After euthanasia, blood was obtained via heart puncture, and serum was isolated and stored at −80 °C. Blood urea nitrogen (control, 15.8939 ± 1.2360 mg/dL; CKD, 71.8677 ± 2.1999 mg/dL) and creatinine (control: 0.3657 ± 0.0264 mg/dL, CKD: 1.9483 ± 0.1573 mg/dL) levels were measured. In addition, kidneys were retained for histological analysis.

### 4.9. Hematoxylin and Eosin (H&E) Staining

At 25 days post melatonin injection with or without the miR-4516 inhibitor, the kidney tissues were removed and fixed with 4% paraformaldehyde (Sigma-Aldrich), and each animal group was embedded in paraffin. For histological analysis, the samples were stained with H&E to study the fibrosis.

### 4.10. Ethics Statement

All animal care procedures and experiments were approved by the Institutional Animal Care and Use Committee of Soonchunhyang University Seoul Hospital (IACUC2013-5, 16 February 2014). Eight-week-old male BALB/c mice (Biogenomics, Seoul, Korea) were used for animal experiments. The mice were maintained on a 12 h light/dark cycle at 25 °C in accordance with the regulations of Soonchunhyang University Seoul Hospital. All experiments were performed in accordance with the National Research Council (NRC) Guidelines for the Care and Use of Laboratory Animals.

### 4.11. Statistical Analysis

All values are represented as the mean ± standard error of the mean (SEM). Statistical significance was analyzed by one-way analysis of variance (ANOVA). Some comparisons of ≥3 groups were made using the Bonferroni–Dunn test. P values < 0.05 were considered to be statistically significant. These data were statistically analyzed using SigmaPlot (Systat Software, San Jose, CA, USA).

## Supplementary Materials

The following are available online at https://www.mdpi.com/1422-0067/21/15/5323/s1. Figure S1. Effect of melatonin on cell cycle and apoptosis of *P*-cresol-treated TH1 cells. Figure S2. Melatonin inhibitor luzindole blocks the effects of melatonin on mitochondrial function. Figure S3. Effect of melatonin on cell cycle and apoptosis of *P*-cresol-treated TH1 cells.
