# Supplementary material for: Melatonin Suppresses Renal Cortical Fibrosis by Inhibiting Cytoskeleton Reorganization and Mitochondrial Dysfunction through Regulation of miR-4516
Source: Int J Mol Sci. 2020 Jul 27;21(15):5323. doi: 10.3390/ijms21155323 (PMC7432329; doi:10.3390/ijms21155323)
Supplement: Supplementary file 1 [file ijms-21-05323-s001.pdf]

International Journal of
Molecular Sciences

Supplementary Figures

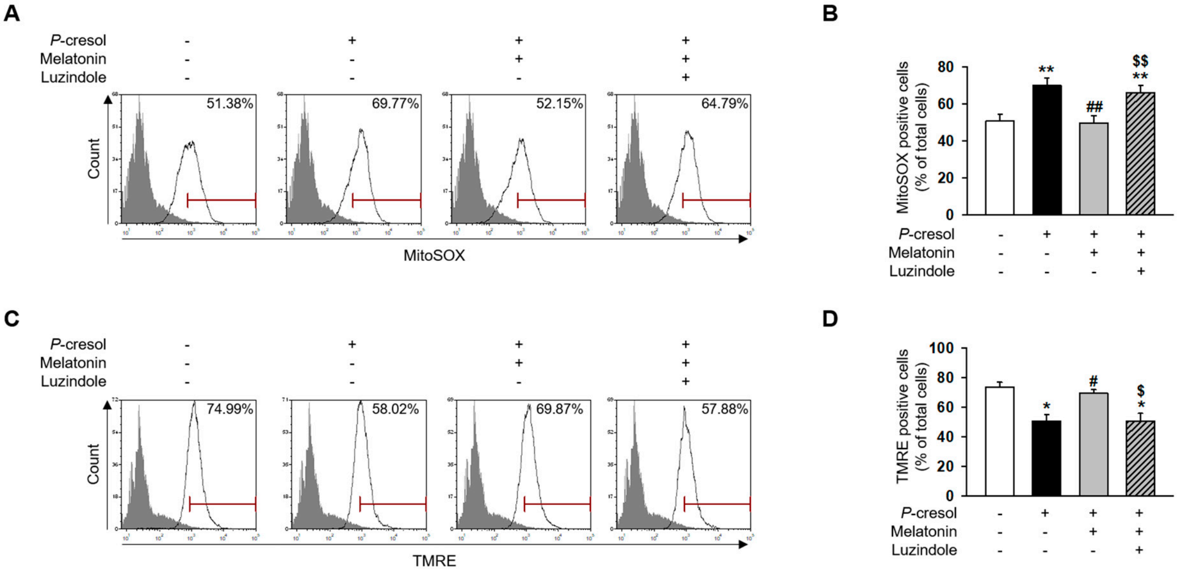

Figure S1. Melatonin inhibitor luzindole blocks the effects of melatonin on mitochondrial ROS generation and mitochondrial membrane potential. (**A**) Flow cytometry analysis of MitoSOX in melatonin-treated TH1 cells (1 μM for 24 h) or cells pre-treated with the luzindole (1 μM for 48 h) under *P*-cresol exposure (0.5 mM for 72 h) (n = 3). (**B**) Quantification of the number of MitoSOX-positive cells. (**C**) Flow cytometry analysis of TMRE in melatonin-treated TH1 cells (1 μM for 24 h) or cells pre-treated with the luzindole (1 μM for 48 h) under *P*-cresol exposure (0.5 mM for 72 h) (n = 3). (**D**) Quantification of the number of TMRE-positive cells. The values represent mean ± SEM. $^{*}p < 0.05$, $^{**}p < 0.01$ vs. control, $^{\#}p < 0.05$, $^{\#\#}p < 0.01$ vs. *P*-cresol exposure condition, $^{\$}p < 0.05$, $^{\$\$}p < 0.01$ vs. melatonin-treated TH1 cells following *P*-cresol exposure.

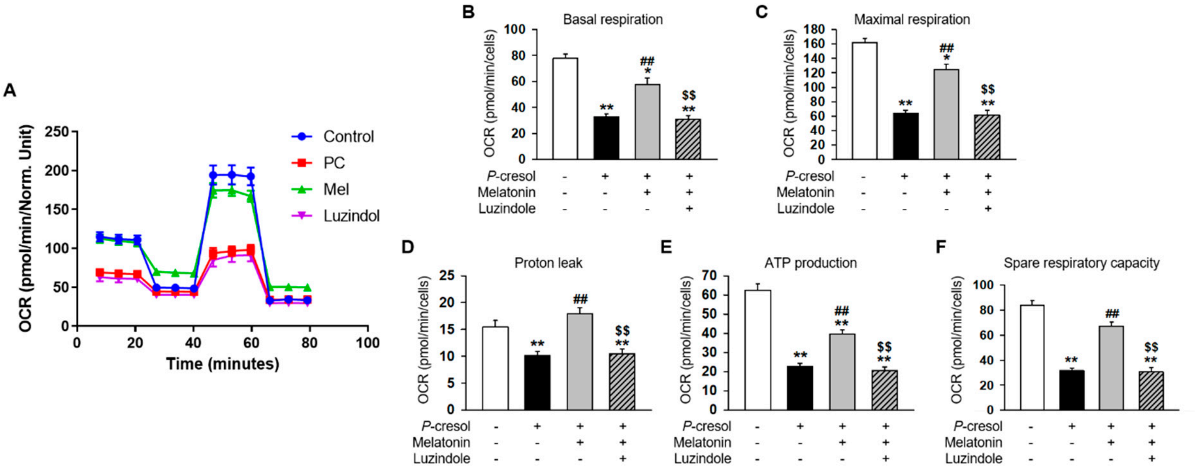

Figure S2. Melatonin inhibitor luzindole blocks the effects of melatonin on mitochondrial function. (**A**) OCR of each group was measured over time (min). The mitochondrial OCR was used to obtain bioenergetic parameters by adding oligomycin (20 min, 1 μM), FCCP (40 min, 0.75 μM), and antimycin A (60 min, 1 μM). (**B**) The non-mitochondrial OCR was subtracted to obtain the basal mitochondrial OCR (remaining OCR after antimycin A addition). (**C**) FCCP was used to obtain the maximal respiration OCR. (**D**) Proton leak was calculated using the difference between OCR following oligomycin A inhibition and OCR following antimycin A inhibition. (**E**) ATP production was determined using the difference between the basal and antimycin A inhibited OCR. (**F**) Spare respiratory capacity was calculated using the difference between the OCR following oligomycin A inhibition and the OCR following FCCP treatment. The histogram shows representative data from one replicate experiment ($n = 5$). The values represent mean ± SEM. *$p < 0.05$, **$p < 0.01$ vs. control, $^{\#\#}p$ < 0.01 vs. *P*-cresol exposure condition, $^{\$\$}p < 0.01$ vs. melatonin-treated TH1 cells in following *P*-cresol exposure.

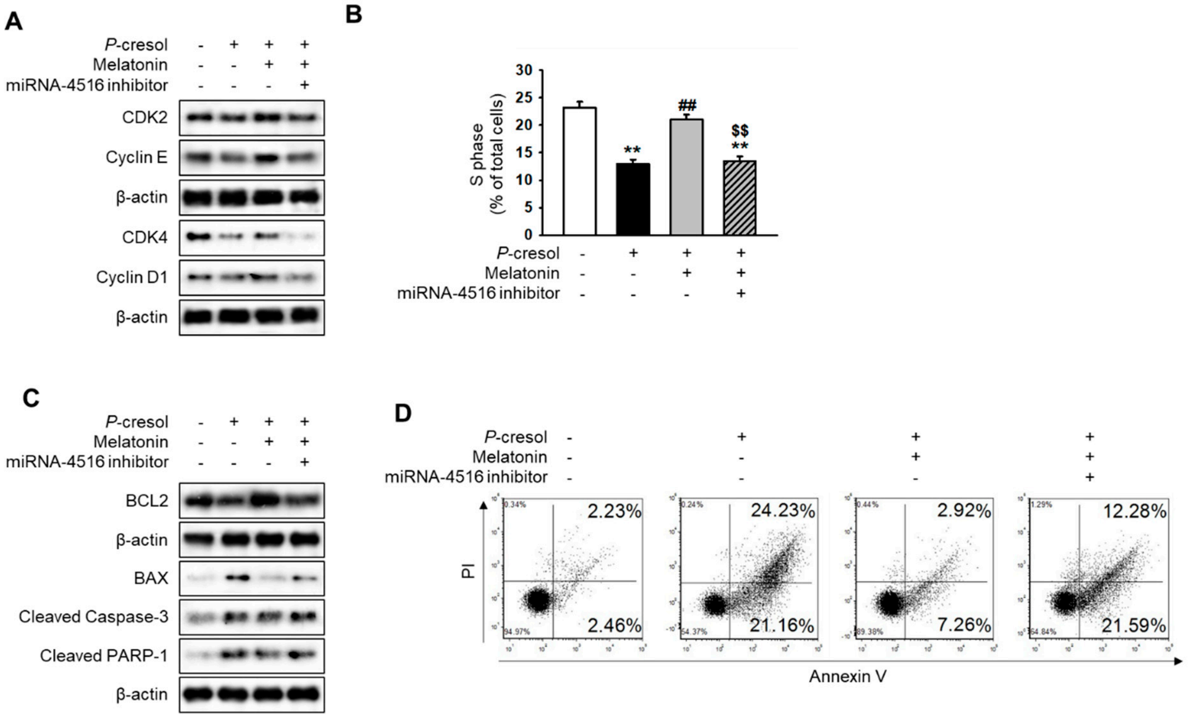

Figure S3. Effect of melatonin on cell cycle and apoptosis of *P*-cresol-treated TH1 cells. (**A**) Western blot analysis for CDK2, cyclin E, CDK4, and cyclin D1 in melatonin-treated TH1 cells (1 μM for 24 h) or cells pre-treated with luzindole (1 μM for 48 h) or the miR-4516 inhibitor (200 nM for 48 h) under

P-cresol exposure (0.5 mM for 72 h) ($n = 3$). (**B**) S-phase flow cytometry analysis in melatonin-treated TH1 cells (1 μM for 24 h) or cells pre-treated with luzindole (1 μM for 48 h) or the miR-4516 inhibitor (200 nM for 48 h) under *P*-cresol exposure (0.5 mM for 72 h) ($n = 3$). The values represent mean ± SEM. $^{**}p < 0.01$ vs. control, $^{\#\#}p < 0.01$ vs. *P*-cresol exposure condition, $^{\$\$}p < 0.01$ vs. melatonin-treated TH1 cells in *P*-cresol exposure condition. (**C**) Western blot analysis for BCL2, BAX, cleaved caspase-3, and cleaved PARP-1 in melatonin-treated TH1 cells (1 μM for 24 h) or cells pre-treated with luzindole (1 μM for 48 h) or the miR-4516 inhibitor (200 nM for 48 h) under *P*-cresol exposure (0.5 mM for 72 h) ($n = 3$). (**D**) Apoptosis in TH1 cells was analyzed by annexin V/PI staining.

© 2020 by the authors. Licensee MDPI, Basel, Switzerland. This article is an open access article distributed under the terms and conditions of the Creative Commons Attribution (CC BY) license (http://creativecommons.org/licenses/by/4.0/).
